# The Complexity of Pain Management in Children Affected by Mucopolysaccharidoses

**DOI:** 10.1155/2017/7257230

**Published:** 2017-04-03

**Authors:** Sabrina Congedi, Chiara Di Pede, Maurizio Scarpa, Angelica Rampazzo, Franca Benini

**Affiliations:** ^1^Pediatric Pain and Palliative Care Service, Department of Women's and Children's Health, University of Padova, Via Giustiniani 3, 35128 Padova, Italy; ^2^Pediatric Pain and Palliative Care Service, Padova, Italy; ^3^Laboratory of Diagnosis and Therapy of Lysosomal Disorders, Department of Women's and Children's Health, University of Padova, Via Giustiniani 3, 35128 Padova, Italy

## Abstract

Mucopolysaccharidoses (MPSs) are a group of rare, genetic lysosomal storage disorders. They are caused by deficiencies of the lysosomal enzymes involved in the degradation of glycosaminoglycans (GAGs). Pain is a common feature in mucopolysaccharidoses. However, the pathophysiology of pain in this group of diseases is still unclear and genesis of pain is multifactorial. Currently, poor data about pain management in these patients are available. Here, we present our clinical experience in complex pain management in three children with MPS.

## 1. Introduction

Mucopolysaccharidoses (MPSs) are rare, heterogeneous, metabolic disorders due to deficiency in the enzymatic degradation of the complex carbohydrate. The incidence of the disease is 1 : 25.000 [1] and the prevalence 2–3,5 per 100.000 live births [1–5]. They are caused by the deficiency in the enzymatic degradation of the complex carbohydrate, called glycosaminoglycans (GAGs). In the 7 types of MPSs (I, II, III, IV, VI, VII, and IX) 11 different enzyme deficiencies have been identified. GAGs incompletely degraded accumulate in multiple site altering cellular, tissue, and organ homeostasis [6]. Patients' presentation at birth is generally normal; after the progressive GAG storage, clinical manifestations start. Moreover, disease severity, phenotype, and rate of progression could be very different for each MPS type [6].

A MPS patient can develop many clinical manifestations: facial dysmorphism, joint and bone disease, ear and upper airway recurrent infections, ophthalmological involvement, hearing loss, cardiorespiratory disorders, dental abnormalities, gastrointestinal problems, neurological damage and, in some cases, neurocognitive impairment [7]. Musculoskeletal system is frequently damaged in MPSs I, II, III, VI, and VII [8]: bone tissue, articular cartilage, and surrounding connective tissue can be involved, as manifestations of GAG storages [9].

Many authors reported clinical manifestations suggesting pain experience in MPS patients [7, 10–13]. With involvement of multisystemic disease, the etiopathogenesis of pain is multifactorial. However, poor information is available about the pathology and management of pain in this group of diseases.

Rarity of the disease, many clinical manifestations, and patients' cognitive delay are the major causes of inadequate pain management in MPS children.

## 2. Case  1

**Figure 1:**
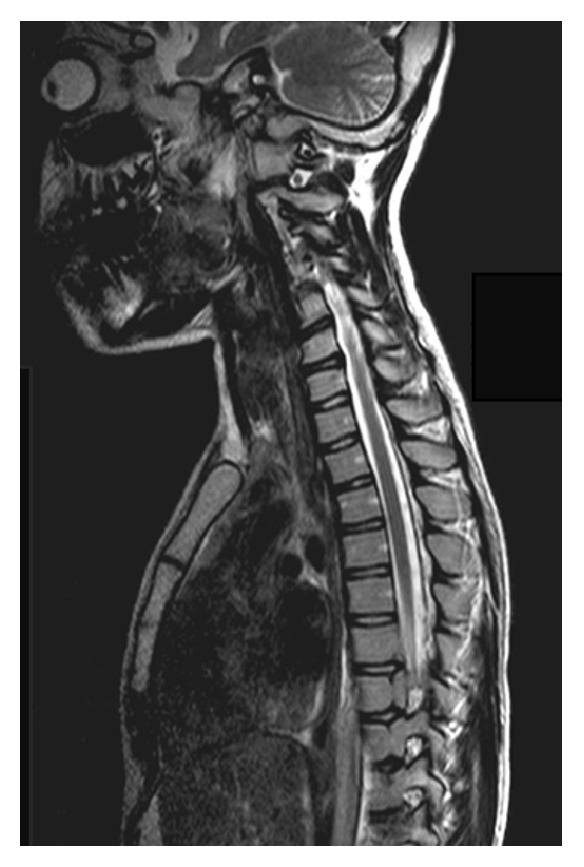
Rachis MRI detecting the presence of GAG around the dens of the epistropheus and the anomalies of spinal canal's diameter.

A 12-year-old girl with MPS3B was referred to our Pediatric Pain Service for an antalgic evaluation of a pain that started 3 months ago. She refused to keep sitting position and she slept with neck retraction and arching of the back, without any preference for a side. A previous event of low back pain was reported in her medical history successfully treated with anti-inflammatory drugs. In this occasion, a rachis MRI evidenced GAG storages around the dens of the epistropheus and diameter reduction of upper cervical spinal canal (Figure 1).

At admission to our Unit she was irritable and suffering; her verbal production and relation with the interlocutor were absent, due to her severe cognitive impairment.

**Figure 2:**
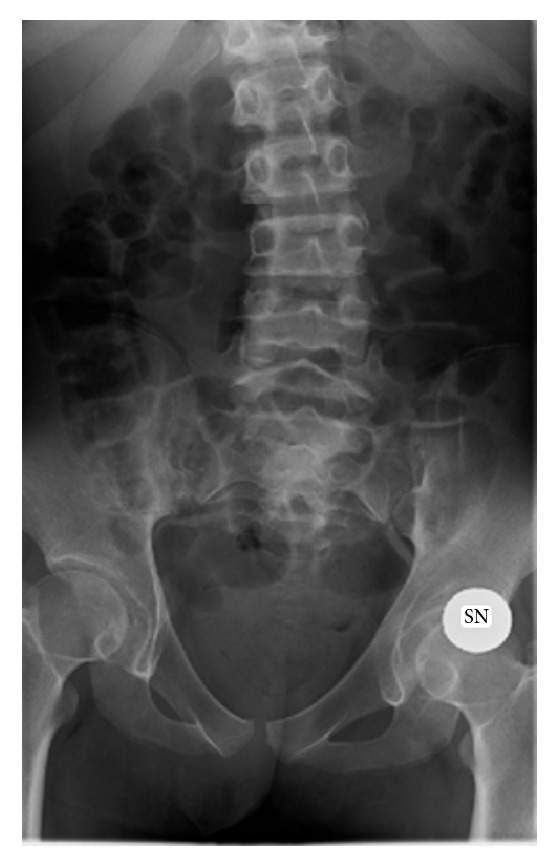
Dorsal scoliosis.

At inspection, limbs and head stereotyped movements and a convex right dorsal scoliosis (Figure 2) were observed. On examination, Vallex points and Lasègue's sign were negative bilaterally; Wasserman test was positive on the left side. Passive extension of hips caused patient's vocalism and laments that disappeared in the lateral decubitus position with flexed thighs. Pain was not accentuated by acupressure of spinal apophysis by palpation of paravertebral muscles of lumbosacral spinal cord nor by passive flexion of the hips. Pain intensity was exacerbated by sitting position that was totally refused by the girl and elicited her crying, while upright position and deambulation with supports were well tolerated. An unsteady march and a right asymmetry in load carrying were observed. On the basis of clinical history and physical examination we diagnosed a persistent left cruralgia. Clinical history, physical examination, biochemical exams, and medical consultations allowed us to exclude other causes of pain (toothache, gastroesophageal reflux, abdominal pathologies, and respiratory and urinary infections). Pain intensity was 7/10 on r-FLACC scale, a validated tool for measurement of pain in disabled children.

After a treatment with indomethacin (1,7 mg/kg/day for 10 days, po) and gabapentin (different doses for 15 days up to 13,6 mg/kg/die and progressively suspended) no pain was measured (0/10 on r-FLACC scale).

## 3. Case  2

In 2014 an 18-year-old girl with MPS IV, coming from the southeast of Italy, was referred to our Pediatric Pain Service for chronic low back pain and cephalalgia, both interfering with sleep and daily activities and partially responding to pain-relieving drugs. Frontal cephalalgia was described as stabbing; photosensitivity, phonophobia, nausea, and vomit were absent. She complained from a stinging low back pain reduced by the left lateral decubitus position.

On admission the girl was suffering; discomfort while sitting in her wheelchair was evident; her cephalalgia and low back pain intensity were both 8-9/10 NRS (numeric rating scale). On physical examination, she presented scaphocephaly, short neck, and convex right dorsal and convex left lumbar scoliosis, with an accentuated reduction of lumbar lordosis and deformity of the sternum. Generalized muscle hypotonia and moderate muscular weakness were present. At inspection, straight cervical rachis was evident; it was associated with severe limitation of range of motion (ROM); no pain was reported during active and passive mobilization at that level. Back pain was raised by acupressure of dorsal and lumbar spinal apophysis and palpation of right lumbar paravertebral muscles. Retraction of hip flexors and hamstring muscles was present. Neither irradiation to lower limbs nor paresthesias were present. Blood phlogosis markers were not altered. Brain and rachis MRI showed many anomalies: cortical atrophy and gliosis in frontal and temporal sites, diameter reduction of spinal canal* at* C1–D7 levels (hourglass aspect of lower brainstem and upper spinal cord), diffuse herniated disks with spinal cord compression, and L5-S1 anterior spondylolisthesis.

On admission, patient's therapy was the following: morphine (0,3 mg/kg × 4/day, po) and betamethasone (0,017 mg/kg × 2/day, po), started 6 months ago. Lateral rachis radiography and DXA (Dual Energy X-ray absorptiometry) were performed to exclude vertebral fractures. After our examination morphine dosage was increased to 0,4 mg/kg × 4/day while betamethasone was continued at the same dose. Pain monitoring was performed thanks to scheduled phone calls. After few weeks, while low back pain intensity decreased, cephalalgia worsened.

For this reason she referred to a pain center near her home; in that occasion, suspecting a left trigeminal neuralgia due to GAG accumulation, steroid and anaesthetic subcutaneous infusions administered locally (once a week, for 3 months) were performed without success. Corticosteroid and morphine therapy were continued.

On her second admission to our service in March 2015, she continued to report severe cephalalgia (NRS 9/10) with hyperalgesia in left periorbital site and moderate low back pain (5-6/10) with paresthesia in the legs. Morphine (0,5 mg/kg × 6/day, po) was associated with gabapentin (up to 10 mg/kg × 3/day, po), while betamethasone was suspended. After one month, the patient reported a reduction of pain in periorbital site (NRS from 9/10 to 4/10).

A new antalgic evaluation was requested in March 2016: low back pain and cephalalgia were still present despite the consumption of morphine for a long time. In this occasion morphine was stopped and Fentanyl patch (1,7 mcg/kg/h every 72 hours) was started suggesting a supplementary Fentanyl transmucosal dose (400 mcg) at need. Amitriptyline and gabapentin were also recommended to treat the neuropathic component of pain but also this pharmacological approach failed. During treatment, the patient presented side effects (difficult urination, nausea, and drowsiness) related to opioid use. At this point, considering the poor analgesic efficacy of morphine and the severe side effects, a cannabinoid therapy was started: 8 mg/kg × 2/die in decoction. After few weeks cephalalgia and opioid side effects vanished. However, she continued assumption of morphine at need (10 mg per os) and betamethasone (0.25 mg/die). We monitored her pain by phone: 1 month and 2 months after the beginning of cannabinoid use, well-being and no side effects were reported.

## 4. Case  3

A 12-year-old boy with MPS-3A and severe cognitive impairment was referred by Pediatric Neurologists to our Pediatric Pain Service for irritability of unknown origin.

**Figure 3:**
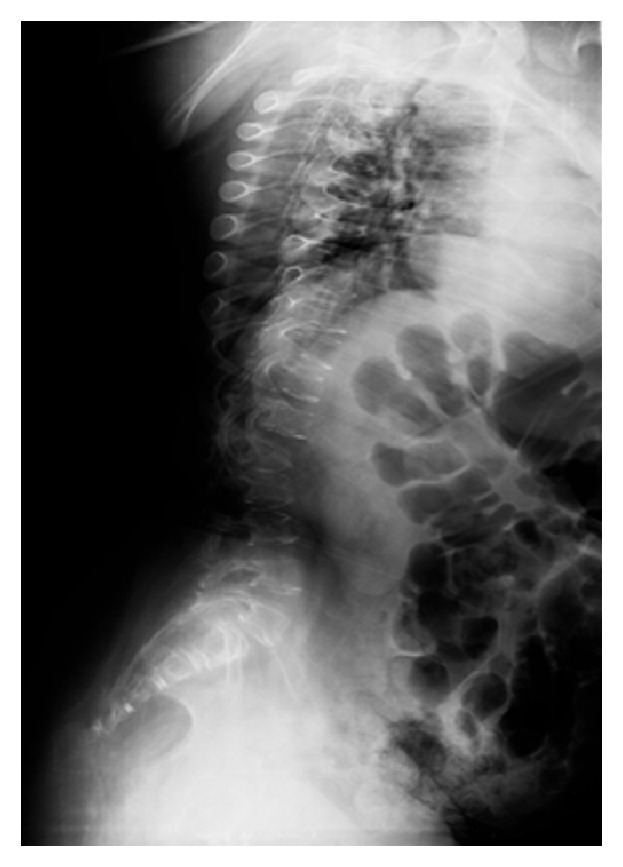
Straightening of rachis's curve.

On examination, a typical face, a sagittal scoliosis with straight physiological curves of the rachis (Figure 3), tetraplegia with generalized muscle weakness, and hypertonia localized to arms with no trunk and head control were observed. During passive mobilizations of arms, we observed face grimaces, irregular breath, moans, and whimpers; joint stiffness was also detected. These elements strongly suggested the presence of nociceptive joint pain; bone fractures were also excluded. Pain intensity on r-FLACC scale was 6/10. Acetaminophen was administered (15 mg/kg × 2/die for 7 days) with pain resolution (0/10 r-FLACC scale).

## 5. Discussion

We presented three examples of complex pain management in children affected by MPS. After carrying out a literature research, we can point out that, despite the burden of pain in MPS children, few data are available concerning pain physiopathology and management in MPS children.

In our experience a mixed pain, both nociceptive and neuropathic, was present in 2 cases, and a pure nociceptive pain was detected in one case. Previous studies reported musculoskeletal problems (bone and joint manifestations, skeletal deformities) as principal features of MPSs types I, II, III, VI, and VII [8], both in the early-stage and during the course of the disorder [6, 12, 14]. Many studies reported musculoskeletal problems as frequent causes of pain in MPSs children (Table 1). GAG storages activated inflammation and it caused tissue damage pain. However, no works focused on pain assessment, diagnosis, and management in children affected by MPSs.

In our patients pain assessment was requested during the course of illness and the somatic nociceptive component of pain has been described in MPS III/IV background.

In our patients pain had also a neuropathic origin. Carpal tunnel syndrome (CTS) has been reported as a frequent cause of neuropathic pain in MPS children [11, 15–18].

No extensive data are available regarding other examples of neuropathic pain in MPSs patients.

In our first case, a double drug therapy was successfully used: indomethacin and gabapentin. The rationale for use of indomethacin, a nonsteroidal anti-inflammatory drug, in the treatment of nociceptive pain component is supported by its anti-inflammatory activity through the nonselective inhibition of COX. It is usually used to relieve symptoms of musculoskeletal conditions, such as arthritis and other joint inflammatory conditions [19]. Since a mixed pain was diagnosed gabapentin, a drug similar to GABA, was also used. In the treatment of neuropathic pain, it is recommended only in the adult population, while its use in the pediatric population is still off-label.

Opioids, corticosteroid, and gabapentin were largely used in the second case. Management of pain in this patient was the most complex and challenging, due to the presence of two different types of chronic pain in the same person. Different pharmacological approaches failed and cannabinoids represented the last available solution. In literature, few works are available regarding risks and benefits of cannabinoids for chronic pain in adults, and there are even less data for adolescents. Despite benefits reported, adverse effects that impair daily activities strongly reduce their use (impaired concentration, lengthened reaction time) [20].

In the third case a pure nociceptive pain was diagnosed and successfully treated with acetaminophen, for its pain reliever activity. These examples confirm the burden of pain in MPS children. Therefore, it is paramount to conduct an adequate assessment, measurement, and diagnosis of the pain. Unavoidable steps should be followed by the pain team to reach a successful pain management.

Pain characteristics such as timing, situation of onset, provocation and palliation, intensity, quality, region and radiation, drug administration, and drug response should be always evaluated for a correct pain management. Cognitive impairment should be considered to choose the correct pain assessment tool. There is no doubt that individuals who are not able to self-report their pain represent a great challenge in pain assessment and a significant barrier to effective pain management. The complexity for health-workers increases when the patient unable to self-report pain is a child.

Moreover disabled children experience pain more frequently than healthy contemporaries [21] and pain is more frequent in MPSs patients with a cognitive delay. Therefore, validated pain scale for children with severe intellectual delay should be used. In our clinical practice, we used the r-FLACC scale, an efficacious, accurate, and reliable tool. This pain scale should be used for 4–19-year-old children with cognitive impairment and it includes general and specific behavioural descriptors [22]. It should be built for each child by a parent or a care-giver with a detailed discussion, collecting child's features about all five scale items (face, leg, activity, cry, and consolability). Parent or care-giver identifies child behaviours that are present in painful and healthy situations.

Many efforts should be made to improve pain evaluation and management to guarantee the best quality of life to children and their families.

## 6. Conclusions

Our clinical experience confirms the complex management of pain in children affected by MPS. Poor data are available regarding this topic. More resources should be assigned to research to better understand pathogenesis of pain in MPS and develop new specific molecules; however an adequate pain assessment is the first step to guarantee a good pain management.

## Figures and Tables

**Table 1:** Experiences about pain in children with MPS.

Source	Subjects	MPS type	Prevalence of joint pain
Brans et al. [10]	89 adult and pediatric MPS patients (55 of whom agreed to participate)	MPS I, MPS II, MPS III, MPS IV, MPS VI, MPS type unknown	69% of children reported joint pain, mainly hip (27,8%) and back pain (25,9%). The highest frequency of pain was observed in MPS III group (52.9%)

Hendriksz et al. [23]	Adult and pediatric MPS patients with	Morquio A Syndrome (MPS IVA)	64% of children reported joint pain (spinal area (63%), lower extremities (100%), upper extremities (69%), and head and neck area (56%))

Vijay and Wraith [11]	29 adult and pediatric MPS patients	Attenuated MPSI phenotype	Progressive arthropathy (86%), fixed flexion deformity of the fingers (24%), and kyphosis, scoliosis, and/or lordosis (24%)

White and Sousa [12]	18 pediatric MPS patients	MPSIII	Many patients requested orthopaedic evaluation of hip pain (hip dysplasia in 8 patients; bilateral osteonecrosis of the femoral heads in 4 patients)

de Ruijter et al. [13]	33 adult and pediatric MPS III patients	MPS-3A, MPS-3B, MPS-3C	For 15 of the 33 patients, pain was indicated in one or both hips

## Abbreviations

MPSs: Mucopolysaccharidoses
GAGs: Glycosaminoglycans
NRS: Numeric rating scale
CTS: Carpal tunnel syndrome.
